# An Accelerated Proximal Gradient Algorithm for Singly Linearly Constrained Quadratic Programs with Box Constraints

**DOI:** 10.1155/2013/246596

**Published:** 2013-10-07

**Authors:** Congying Han, Mingqiang Li, Tong Zhao, Tiande Guo

**Affiliations:** ^1^School of Mathematical Sciences, University of Chinese Academy of Sciences, Shijingshan District, Beijing 100049, China; ^2^College of Information Science and Engineering, Shandong University of Science and Technology, Qingdao 266590, China

## Abstract

Recently, the existed proximal gradient algorithms had been used to solve non-smooth convex optimization problems. As a special nonsmooth convex problem, the singly linearly constrained quadratic programs with box constraints appear in a wide range of applications. Hence, we propose an accelerated proximal gradient algorithm for singly linearly constrained quadratic programs with box constraints. At each iteration, the subproblem whose Hessian matrix is diagonal and positive definite is an easy model which can be solved efficiently via searching a root of a piecewise linear function. It is proved that the new algorithm can terminate at an *ε*-optimal solution within $O(1/\sqrt{\varepsilon })$ iterations. Moreover, no line search is needed in this algorithm, and the global convergence can be proved under mild conditions. Numerical results are reported for solving quadratic programs arising from the training of support vector machines, which show that the new algorithm is efficient.

## 1. Introduction

 In this paper, we mainly consider the following quadratic programming problem:

(1) $$\begin{array}{c} \underset{x\in \Omega }{\min}f\left(x\right)=\frac{1}{2}x^{T}Gx-b^{T}x, \end{array}$$

where *G* ∈ *R* ^*n*×*n*^ is symmetric and positive semidefinite, *x*, *b* ∈ *R* ^*n*^, and the feasible region *Ω* is defined by

(2) $$\begin{array}{c} \Omega =\left\{x\in R^{n},l\leq x\leq u,c^{T}x=d\right\}, \end{array}$$

where *l*, *u*, *c* ∈ *R* ^*n*^ and *d* is a scalar. 

 The singly linearly constrained quadratic programs with box constraints appear in a wide range of applications such as image processing, biological information and machine learning. Specifically, support vector machine (SVM) is one of the most classical models of (1). It is a promising technique for solving a variety of machine learning and function estimation problems. The SVM learning methodology has been shown to give good performance in a wide variety of problems such as face detection, text categorization, and handwritten character recognition. The number of variables in SVM is so huge that traditional optimization methods cannot be directly applied. Some decomposition method [1–4] with its subproblem being a special case of (1) is the main approach for large-scale SVM problems. The solution of the subproblem by generalized variable projection method (GVPM) and projected gradient method (PGM) is introduced in [5, 6], respectively. Moreover, Zanni, and so forth, also proposes the parallel decomposition algorithms based on these two methods in [7, 8]. For more general large-scale model, some of the parallelization pieces of literature in [9, 10] recently are proposed, but these methods cannot be applied specifically to SVM so far. 

In this work, we will give an accelerated proximal gradient algorithm for (1). First, we consider the following nonsmooth convex optimization problem:

(3) $$\begin{array}{c} \underset{x\in R^{n}}{\min}F\left(x\right)=h\left(x\right)+g\left(x\right), \end{array}$$

where *g* : *R* ^*n*^ → (−*∞*, *∞*] is a proper, lower semicontinuous (lsc), convex function and *h* is convex smooth (i.e., continuously differentiable) on an open subset of *R* ^*n*^ containing dom *g* = {*x* | *g*(*x*) < *∞*}.  ∇*h* is Lipschitz continuous on dom *g*. That is,

(4) $$\begin{array}{c} ||\nabla h\left(x\right)-\nabla h\left(y\right)||\leq L||x-y||\;\forall x,y\in \mathrm{dom}g \end{array}$$

for some *L* > 0, where, and in what follows, ||·|| denotes the spectral norm. Obviously, problem (1) is a special case of (3) with *h*(*x*) = *f*(*x*) and *g*(*x*) being the indicator function for the feasible region *Ω* defined by

(5) $$\begin{array}{c} g\left(x\right)=\{\begin{array}{cc} 0 & \text{if}\;x\in \Omega ,\\ +\infty & \text{else}. \end{array} \end{array}$$

Recently, great attention has been paid to the solution of (3). Nesterov and Nemirovski [11, 12] study the accelerated proximal gradient method for (1) with an attractive iteration complexity of $O(1/\sqrt{\varepsilon })$ for achieving *ε*-optimality. Almost at the same time, Beck and Teboulle [13] give a fast iterative shrinkage-thresholding algorithm (FISTA) which achieves the same convergence rate. After that, Tseng [14] summarizes these algorithms and presents a unified treatment of these methods. All these algorithms have a good performance on large-scale problems, such as linear inverse problems, matrix game problems, and matrix completion. Motivated by the successful use of the accelerated proximal gradient method for (3), we extend Beck and Teboulle's algorithm to solve (1). In particular, the subproblem is solved by searching a root of a piecewise linear continuous function. Numerical results show that the new algorithm is efficient. 

 The paper is organized as follows. In Section 2, the proximal gradient algorithm and its accelerated version are presented for (1). The convergence of these algorithms is also discussed. In Section 3, the method to efficiently solve the subproblem is introduced. Numerical results on SVM classification problems generated by the random and real world data sets are shown in Section 4.  Section 5 is devoted to some conclusions and further study. 

## 2. Proximal Gradient Algorithms

 In this section, we introduce the proximal gradient algorithm and its accelerated version which can be applied to solve (1). For any *z* ∈ *R* ^*n*^, consider the following quadratic approximation of *f*(*x*) at *z*:

(6) QL(x,z)=f(z)+〈x−z,∇f(z)〉+L2||x−z||2

(7) =L2xTx+(Gz−Lz−b)Tx+const,

where *L* > 0 is a given parameter, const = (*L*/2)*z* ^*T*^ *z* − ∇*f*(*z*)^*T*^ *z* + *f*(*z*). Since (6) is a strongly convex function of *x*, *Q*_*L*_(*x*, *z*) has a unique minimizer in *Ω* which we denote by

(8) $$\begin{array}{c} P_{L}\left(z\right)=\text{argmin}\left\{Q_{L}\left(x,z\right):x\in \Omega \right\}. \end{array}$$

First, we present a simple result which characterizes the optimality of *P*_*L*_(*z*).

**Lemma**

For any *z* ∈ *R* ^*n*^, *w* = *P*_*L*_(*z*) if and only if there exists *γ*(*z*) ∈ *N*_*Ω*_(*w*), such that

(9) $$\begin{array}{c} \nabla f\left(z\right)+L\left(w-z\right)+\gamma \left(z\right)=0, \end{array}$$

where *N*_*Ω*_(*w*) is the normal cone of *Ω* at the point *w* denoted by

(10) $$\begin{array}{c} N_{\Omega }\left(w\right)=\left\{w^{*}\in R^{n}\;|\;\langle w^{*},v-w\rangle \leq 0\;\forall v\in \Omega \right\}. \end{array}$$

**Proof**

This result can be obtained directly from the optimality conditions of (8), which is a strongly convex problem.

Then, similarly to Lemma 2.3 in [13], we have the following key result.

**Lemma**

Let *z* ∈ *R* ^*n*^ and *L* > 0 such that

(11) $$\begin{array}{c} f\left(P_{L}\left(z\right)\right)\leq Q\left(p_{L}\left(z\right),z\right). \end{array}$$

Then for any *x* ∈ *Ω*,

(12) $$\begin{array}{c} f\left(x\right)-f\left(P_{L}\left(z\right)\right)\geq \frac{L}{2}{||P_{L}\left(z\right)-z||}^{2}+L\langle z-x,P_{L}\left(z\right)-z\rangle . \end{array}$$

**Proof**

Since *γ*(*z*) ∈ *N*_*Ω*_(*w*) and *f* is convex, it follows that

(13) $$\begin{array}{c} f\left(x\right)\geq f\left(z\right)+\langle x-z,\nabla f\left(z\right)\rangle +\langle \gamma \left(z\right),x-P_{L}\left(z\right)\rangle . \end{array}$$

Hence, from (9), (11), (13), and the definition of *P*_*L*_(*z*) we have

(14) f(x)−f(PL(z)) ≥f(x)−Q(pL(z),z) ≥−L2||PL(z)−z||2+〈x−PL(z),∇f(z)+γ(z)〉 =−L2||PL(z)−z||2+L〈x−PL(z),z−PL(z)〉 =L2||PL(z)−z||2+L〈z−x,PL(z)−z〉.

This completes the proof.

Note that *f*(*x*) is a convex quadratic function and (11) can always be satisfied for *P*_*L*_(*z*) if *L* ≥ *λ*_max⁡_(*G*), where *λ*_max⁡_ denotes the maximum eigenvalue of *G*. The proximal gradient algorithm for (1) is as follows.

**Algorithm**

We have the following steps.

*Step 1.* Choose *x*_0_ ∈ *R* ^*n*^.

*Step 2*. While (*x*_*k*−1_ does not satisfy the terminal conditions)

(15) $$\begin{array}{c} x_{k}=P_{L}\left(x_{k-1}\right)\\ k=k+1. \end{array}$$

The following theorem is about the iteration complexity of Algorithm 3. In what follows, X* and f* denote the set of optimal solutions and optimal value of (1), respectively.

**Theorem**

Let {*x*_*k*_} and {*f*(*x*_*k*_)} be the sequences generated by Algorithm 3 with *L* ≥ *λ*_max⁡_(*G*); then, for *k* ≥ 1, the following results hold:

(a) {*f*(*x*_*k*_)} is nonincreasing and
  (16) $$\begin{array}{c} f\left(x_{k}\right)-f\left(x_{k+1}\right)\geq \frac{L}{2}{||x_{k}-x_{k+1}||}^{2}; \end{array}$$
(b) *f*(*x*_*k*_) − *f*(x*) ≤ *L*||*x*_0_−x*||^2^/2*k*, for all x* ∈ X*;
(c) if {*x*_*k*^*j*^_} is a convergent subsequence of {*x*_*k*_} and lim⁡_*j*→*∞*_⁡*x*_*k*^*j*^_ = x** then x** ∈ X*;
(d) if *G* is positive definite, then
  (17) $$\begin{array}{c} ||x_{k}-x^{*}||\leq \sqrt{\frac{L}{2\Lambda_{\min}\left(G\right)k}}||x_{0}-x^{*}||. \end{array}$$

**Proof**

(a) Let *z* = *x*_*k*_, *x*_*k*+1_ = *P*_*L*_(*z*); then from (11) and the optimality of *P*_*L*_(*z*) we have

(18) $$\begin{array}{c} f\left(x_{k+1}\right)\leq Q\left(x_{k+1},x_{k}\right)\leq Q\left(x_{k},x_{k}\right)=f\left(x_{k}\right). \end{array}$$

Furthermore, take *x* = *x*_*k*_ in (12); we get

(19) $$\begin{array}{c} f\left(x_{k}\right)-f\left(x_{k+1}\right)\geq \frac{L}{2}{||x_{k}-x_{k+1}||}^{2}. \end{array}$$

(b) We have

(20) k(f(xk)−f(x∗)) =kf(xk)−∑n=0k−1f(xn+1)+∑n=0k−1f(xn+1)−kf(x∗) ≤∑n=0k−1(f(xn+1)−f(x∗)) ≤−∑n=0k−1(L2||xn+1−xn||2−L〈xn−x∗,xn+1−xn〉) =−L2(∑n=0k−1(||x∗−xn+1||2−||x∗−xn||2)) =−L2(||x∗−xk||2−||x0−x∗||2) ≤L2||x0−x∗||2,

where the first inequality uses the fact that {*f*(*x*_*k*_)} is nonincreasing and the second inequality is established by taking *x* = x*, *z* = *x*_*n*_ in (12). Dividing both sides by *k* in the last inequality, we get

(21) $$\begin{array}{c} f\left(x_{k}\right)-f\left(x^{*}\right)\leq \frac{L{||x_{0}-x^{*}||}^{2}}{2k}. \end{array}$$

(c) It follows from (b) that, for all x* ∈ X*,

(22) $$\begin{array}{c} f\left(x_{k^{j}}\right)-f\left(x^{*}\right)\leq \frac{L{||x_{0}-x^{*}||}^{2}}{2k^{j}}. \end{array}$$

Let *j* → *∞* in the above inequality; we have *f*(x**) ≤ *f*(x*). On the other hand, by the definition of x*, we have *f*(x**) ≥ *f*(x*). Hence, *f*(x**) = *f*(x*), which implies that x** ∈ X*.

(d) It follows from the definition of *f*(*x*) that, for all x* ∈ X*,

(23) f(xk)−f(x∗) =∇f(x∗)T(xk−x∗)+(xk−x∗)TG(xk−x∗) ≥(xk−x∗)TG(xk−x∗) ≥λmin⁡(G)||xk−x∗||2,

where the first inequality is established by the definition of x* and the second inequality, with *λ*_min⁡_(*G*) denoting the minimum eigenvalue of *G*, is obtained from the positive definite property of *G*.

From (23) and (b), we get

(24) $$\begin{array}{c} ||x_{k}-x^{*}||\leq \sqrt{\frac{L}{2\lambda_{\min}\left(G\right)k}}||x_{0}-x^{*}||. \end{array}$$

Theorem 4 shows that Algorithm 3 has a result complexity of *O*(1/*k*). Now we introduce a new algorithm with an improved complexity result of *O*(1/*k* ^2^).

**Algorithm**

We have the following steps.

*Step 1.* Choose *z*_1_ = *x*_0_ ∈ *R* ^*n*^, set *k* = 1, *t*_1_ = 1.

*Step2.* While (*x*_*k*−1_ does not satisfy the terminal conditions)

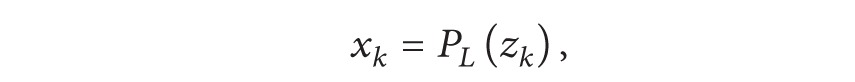
(25)

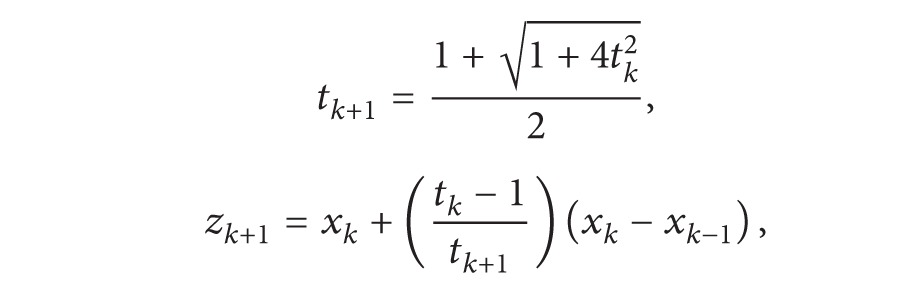
(26)

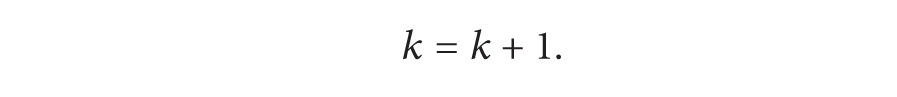
(27)

**Remark 6**

In Algorithm 5, the operator *P*_*L*_(·) is employed on the point *z*_*k*_ which is a specific linear combination of the previous two points {*x*_*k*−1_, *x*_*k*−2_}. However, in Algorithm 3, the operator only uses the previous point *x*_*k*−1_. On the other hand, the computational effort of these two algorithms is almost the same except for the computation of (26) in Algorithm 5 which is negligible.

Now we give the promising improved complexity result for Algorithm 5.

**Theorem**

Let {*x*_*k*_} and {*f*(*x*_*k*_)} be the sequences generated by Algorithm 5 with *L* ≥ *λ*_max⁡_(*G*). Then, for *k* ≥ 1, the following results hold:

(a) *f*(*x*_*k*_) − *f*(x*) ≤ 2*L*||*x*_0_−x*||^2^/(*k* + 1)^2^, for all x* ∈ X*;
(b) if {*x*_*k*^*j*^_} is a converging subsequence of {*x*_*k*_} and lim⁡_*j*→*∞*_⁡*x*_*k*^*j*^_ = x**, then x** ∈ X*;
(c) if G is positive definite, then
  (28) $$\begin{array}{c} ||x_{k}-x^{*}||\leq \sqrt{\frac{L}{2\lambda_{\min}\left(G\right){\left(k+1\right)}^{2}}}||x_{0}-x^{*}||. \end{array}$$

**Proof**

(a) Using Lemma 2, the proof is similar to the proof of Theorem 4.4 in [13]. (b) and (c) can be proved in the same way as that of (c) and (d) in Theorem 4, respectively.

## 3. How to Solve *P*_*L*_(·)

 In this section, we extend the method studied by Dai and Flecher [6] to solve *P*_*L*_(·). In order to obtain *P*_*L*_(·) in (8), we need to solve the following problem:

(29) min⁡⁡ L2xTx−qTx s.t.    cTx=d, l≤x≤u,

where *q* = *Lz* + *b* − *Gz*. For a given value of *λ*, we consider the following box constrained QP problem:

(30) min⁡⁡ L2xTx−qTx−λ(cTx−d) s.t.    l≤x≤u

and denote the minimizer of (30) as *x*(*λ*). Then,

(31) $$\begin{array}{c} x\left(\lambda \right)=\text{mid}\left(l,\alpha ,u\right), \end{array}$$

where *α* = *α*(*λ*) has components *α*_*i*_ = (*q*_*i*_ + *λc*_*i*_)/*L* (*i* = 1,2,…*n*) and mid(*l*, *α*, *u*) is the componentwise operation that supplies the median of its three arguments. That is,

(32) [x(λ)]i=li+αi−ui−max⁡(li,αi,ui) −min⁡(li,αi,ui) (i=1,2,…n).

Define

(33) $$\begin{array}{c} r\left(\lambda \right)=c^{T}x\left(\lambda \right)-d; \end{array}$$

then from [6], we know that *r*(*λ*) is a piecewise linear continuous and monotonically increasing function of *λ*. Furthermore, *x*(λ*) is the optimal of (29) if λ* is located such that *r*(λ*) = 0. Hence, the main task of solving (29) is to find a λ* such that *r*(λ*) = 0. For this purpose, we adopt the algorithm introduced in [6] which consists of a* bracketing phase* and a* secant phase*. Numerical experiments in the next section show that this algorithm achieves high efficiency. 

## 4. Numerical Experiments 

 As we know, one of the important applications of SVM is classification. Given a training set

(34) $$\begin{array}{c} D=\left\{\left(z_{i},y_{i}\right),i=1,\ldots ,n,z_{i}\in R^{m},y_{i}\in \left\{-1,1\right\}\right\}, \end{array}$$

we need to find a hyperplane to separate the two classes of points. This can be done by solving the following convex quadratic programming problem:

(35) min⁡⁡ 12xTQx−eTx s.t.  yTx=0, 0≤x≤Ce,

where *y* = [*y*_1_, *y*_2_,…, *y*_*n*_]^*T*^, *e* ∈ *R* ^*n*^ is the vector of all ones, and *C* is a positive scalar. *Q* is a *n* × *n* symmetric and positive semidefinite matrix with entries *Q*_*ij*_ = *y*_*i*_ *y*_*j*_ *K*(*x*_*i*_, *x*_*j*_), *i*, *j* = 1,2,…, *n*, where *K* : *R* ^*m*^ × *R* ^*m*^ → *R* is some kernel function.

 In this section, we illustrate the performance of Algorithms 3 and 5 with Matlab 7.10 on a Windows XP professional computer (Pentium R, 2.79 GHZ). We conduct a test on SVM classification problems with the random data sets and the real world data sets. 

 The generation of the random data sets is based on four parameters *n*, *m*, *β*, and *η*, where *n* is the number of samples and *m* is the dimension of *z*_*i*_  (*i* = 1,…, *n*). Each element of *z*_*i*_ is randomly generated in [*β*, *η*]  (*i* = 1,…, *n*), and −1 or 1 randomly emerges in the *i*th entry of *y*  (*i* = 1,…, *n*).

We have generated three random data sets with *β* = −1, *η* = 1, *m* = 5, and *n* = 200, 600, and 1000, respectively. The two real world data sets are the UCI adult data set and the heart data set. For the UCI adult, the versions with *n* = 1605, 2265, and 3185, are considered. The Gauss radial basis function

(36) $$\begin{array}{c} K\left(z_{i},z_{j}\right)=\exp\left(-\frac{{||z_{i}-z_{j}||}^{2}}{\sigma^{2}}\right) \end{array}$$

is used in our tests. The parameters *C* in (35) and *σ* in (36) are set to $\left(10,\sqrt{40}\right)$, $\left(1,\sqrt{13}\right)$, and (1, 1000) for random data sets, heart, and UCI adult data sets respectively. 

 For all test problems, the initial point is chosen as *x*_0_ = (0,…0)^*T*^ and the const *L* = ∑_*i*=1_^*n*^ *Q*_*ii*_. The terminal rules for solving *P*_*L*_(·) are |*r*(*λ*)| ≤ 10^−8^. 

**Figure 1:**
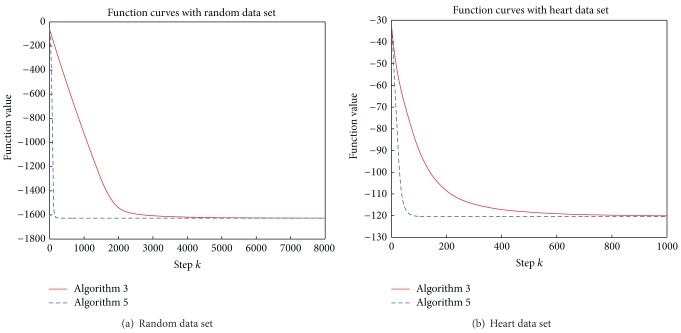
Function value curve with iterations.

**Figure 2:**
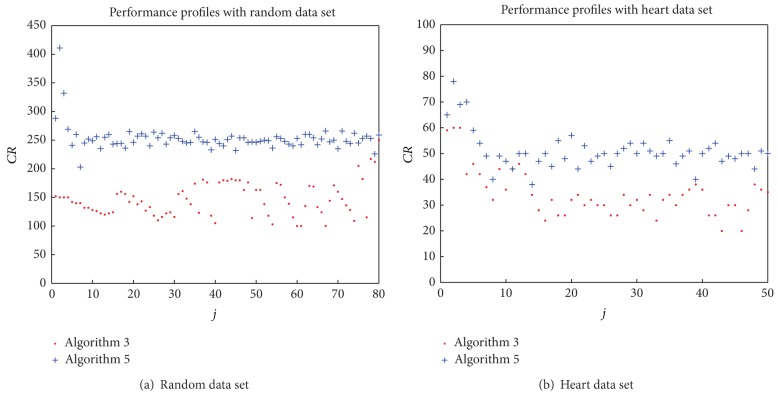
Number of calculating *r*(*λ*) for every finite step.

 For the test problem with random data set (*n* = 200) and heart data set, the running steps are 8000 and 1000, respectively. In order to make a visual comparison between Algorithms 3 and 5, we plot the values of *f*(*x*_*k*_) with iterations in Figure 1. Furthermore, we compute

(37) $$\begin{array}{c} CR\left(j\right)=\overset{M}{\underset{k=1}{\sum }}cr\left(M\times \left(j-1\right)+k\right)\;\left(j=1,2,\ldots J\right), \end{array}$$

where *cr*(*M* × (*j* − 1) + *k*) denotes the number of calculating *r*(*λ*) in step *M* × (*j* − 1) + *k*. In Figure 2, we plot the values of *CR*(*j*)  (*j* = 1,2,…*J*) with *M* = 100, *J* = 80 and *M* = 20, *J* = 50, respectively.

For the test problems with the rest data sets, the terminal condition is based on the fulfilment of the KKT conditions within 0.001 (see [15]). The numerical results are shown in Tables 1 and 2, where the meaning of the indexes are as follows:

- *n*: the scale of the data sets,
- sec: the computing time (in seconds),
- *k*: the number of iterations,
- raver: the average number of calculating *r*(*λ*) in each step.

From Figure 2, we can see that although the total number of calculating *r*(*λ*) every 100 (27) steps in Algorithm 3 is less than that the number in Algorithm 5. However, Algorithm 5 uses much less iterations and time to achieve the approximate solution, compared with Algorithm 3 from Figure 1 and Tables 1 and 2. For the subproblem, less than 4 iterations are used to achieve a very accurate solution on average, which shows that *P*_*L*_(·) can be solved efficiently. Moreover, from the column raver in Tables 1 and 2, we can also see that the average number of calculating *r*(*λ*) with real world data sets is slightly less than that with random data sets. 

## 5. Conclusion and Future Work

 We have extended the accelerated proximal point method to the solution of singly linearly constrained quadratic programming with box constraints. The new algorithm is proved to be globally convergent. Numerical results also show that the new algorithm performs well on medium-scale quadratic programs. On the other hand, Solving the subproblem by searching a root of a piecewise linear continuous function is a very cheap. Considering the good performance of the new Algorithm 5, we can apply it to the solution of the subproblems in decomposition methods for large-scale SVM problems. Moreover, a parallel version of this algorithm combined with the theory in [9, 10] is also a direction of our future research.

## Figures and Tables

**Table 1:** Numerical results for Algorithm 3.

Problems	*n*	Sec	*k*	Raver
Random	600	19.97	19524	2.98
	1000	78.23	32153	2.99

Adult	1605	6.81	1064	1.34
	2265	20.45	1515	1.50
	3185	49.50	2103	1.50

**Table 2:** Numerical results for Algorithm 5.

Problems	*n*	Sec	*k*	Raver
Random	600	5.33	5021	3.68
	1000	19.05	7095	3.30

Adult	1605	1.41	88	2.82
	2265	2.84	106	2.97
	3185	6.30	125	2.98
